# Gender-Specific Differences in Patients With Chronic Tinnitus—Baseline Characteristics and Treatment Effects

**DOI:** 10.3389/fnins.2020.00487

**Published:** 2020-05-25

**Authors:** Uli Niemann, Benjamin Boecking, Petra Brueggemann, Birgit Mazurek, Myra Spiliopoulou

**Affiliations:** ^1^Faculty of Computer Science, Otto von Guericke University Magdeburg, Magdeburg, Germany; ^2^Tinnitus Center, Charité Universitaetsmedizin Berlin, Berlin, Germany

**Keywords:** gender, tinnitus, distress, depression, treatment response, gender-influence on diagnostics, machine learning, variable importance

## Abstract

Whilst some studies have identified gender-specific differences, there is no consensus about gender-specific determinants for prevalence rates or concomitant symptoms of chronic tinnitus such as depression or anxiety. However, gender-associated differences in psychological response profiles and coping strategies may differentially affect tinnitus chronification and treatment success rates. Thus, understanding gender-associated differences may facilitate a more detailed identification of symptom profiles, heighten treatment response rates, and help to create access for vulnerable populations that are potentially less visible in clinical settings. Our research questions are: RQ1: how do male and female tinnitus patients differ regarding tinnitus-related distress, depression severity, and treatment response, RQ2: to what extent are answers to questionnaires administered at baseline associated with gender, and RQ3: which baseline questionnaire items are associated with tinnitus distress, depression, and treatment response, while relating to one gender only? In this work, we present a data analysis workflow to investigate gender-specific differences in *N* = 1,628 patients with chronic tinnitus (828 female, 800 male) who completed a 7-day multimodal treatment encompassing cognitive behavioral therapy (CBT), physiotherapy, auditory attention training, and information counseling components. For this purpose, we extracted 181 variables from 7 self-report questionnaires on socio-demographics, tinnitus-related distress, tinnitus frequency, loudness, localization, and quality as well as physical and mental health status. Our workflow comprises (i) training machine learning models, (ii) a comprehensive evaluation including hyperparameter optimization, and (iii) post-learning steps to identify predictive variables. We found that female patients reported higher levels of tinnitus-related distress, depression and response to treatment (RQ1). Female patients indicated higher levels of tension, stress, and psychological coping strategies rates. By contrast, male patients reported higher levels of bodily pain associated with chronic tinnitus whilst judging their overall health as better (RQ2). Variables measuring depression, sleep problems, tinnitus frequency, and loudness were associated with tinnitus-related distress in both genders and indicators of mental health and subjective stress were found to be associated with depression in both genders (RQ3). Our results suggest that gender-associated differences in symptomatology and treatment response profiles suggest clinical and conceptual needs for differential diagnostics, case conceptualization and treatment pathways.

## 1. Introduction

Tinnitus refers to the perception of sound, such as ringing, whistling, hissing, or rustling in absence of an external sound stimulus (Eggermont and Roberts, 2004) and is experienced by 12–30% of the general population (McCormack et al., 2016). A subset of 1–2% of all people report considerable impairment with respect to quality of life associated with the tinnitus percept (Langguth et al., 2013). Due to its clinical heterogeneity in cause, perception and accompanying symptoms (Langguth et al., 2013), a treatment gold standard that is effective for every patient has not been established yet, despite scientifically proven efficacy of cognitive behavioral approaches in reducing tinnitus-related distress for some patients (Andersson and Lyttkens, 1999; Andersson, 2002; Cima et al., 2012, 2014).

Tinnitus is often accompanied by comorbid symptoms including agitation, anxiety, depression, insomnia, irritability, and stress (Langguth et al., 2009). For example, in between 14 and 80% of tinnitus patients (Langguth et al., 2011), tinnitus is associated with high levels of interacting anxiety and depression levels. Whereas, female gender was identified as one important risk factor of psychological comorbidities in many studies (Piccinelli and Wilkinson, 2000; Nolen-Hoeksema, 2001; Matud, 2004; Jaussent et al., 2011; McLean et al., 2011; Langguth et al., 2013; Asher et al., 2017), the relationship between gender and tinnitus is not well-understood yet. Previous studies have presented contradictory results about the relationship between gender and tinnitus severity and/or distress: Erlandsson et al. (1992) found no difference between genders in tinnitus severity measured by their Tinnitus Severity Questionnaire (TSQ) in 186 tinnitus patients. Pinto et al. (2010) concluded no influence of gender on tinnitus annoyance using the Tinnitus Handicap Inventory (THI; Newman et al., 1996) in 68 tinnitus patients. Further, Meric et al. (1998) found no difference between female and male tinnitus patients (*N* = 281) with respect to the scores of the Tinnitus Handicap Questionnaire (THQ; Kuk et al., 1990), the Tinnitus Reaction Questionnaire (Wilson et al., 1991) and the Subjective Tinnitus Severity Scale (Halford and Anderson, 1991). In contrast, Seydel et al. (2013) reported differences in tinnitus distress measured by the German version of the Tinnitus Questionnaire (TQ; Goebel and Hiller, 1998) between genders in 1,180 tinnitus patients although the manifestation of accompanying symptoms partly depended on age. Hiller and Goebel (2006) found higher levels of tinnitus loudness and annoyance for men measured by the TQ in 4,971 volunteers of the German Tinnitus League (Hiller and Goebel, 2006). Lugo et al. (2019) showed that severe tinnitus was associated with suicide attempts only in women among 71,542 participants of the Stockholm Public Health Cohort (Svensson et al., 2012). Han et al. (2019) revealed that significant associations between tinnitus severity with quality of life, depressivity, and stress prevail only among the male subjects of 248 patients with tinnitus complaints.

Overall, there is no consensus about gender-specific determinants for prevalence rates or concomitant symptoms of chronic tinnitus such as depression or anxiety. However, gender-associated differences in psychological response profiles and coping strategies may differentially affect tinnitus chronification and treatment success rates. Thus, understanding gender-associated differences may facilitate a more detailed identification of symptom profiles, heighten treatment response rates, and help to create access for vulnerable populations that are potentially less visible in clinical settings. In this work, our research questions were: RQ1: how do male and female tinnitus patients differ with respect to tinnitus-related distress, depression severity, and treatment response, RQ2: to what extent are answers to questionnaires administered at baseline associated with gender, and RQ3: which baseline questionnaire items are associated with tinnitus distress, depression, and treatment response, while relating to one gender only? For that purpose, we developed a hypothesis-free data analysis workflow that aimed to identify determinants of gender-specific differences in patients with chronic tinnitus based on data extracted from multiple questionnaires at baseline using machine learning.

## 2. Materials and Methods

### 2.1. Patient Data

This study is based on retrospective data from 4,103 tinnitus patients who had been treated at the Tinnitus Center of Charité Universitaetsmedizin Berlin between January 2011 and October 2015, were 18 years of age or older and had been suffering from tinnitus for at least 3 months. Exclusion criteria were presence of an acute psychotic illness or addiction disorder (abstinence not ensured during treatment), deafness (untreated), and insufficient knowledge of the German language. Treatment comprised multimodal 7-day program that included intensive and daily informational counseling, detailed ear-nose-throat (ENT) as well as psychological diagnostics, cognitive behavior therapy interventions, hearing exercises with aspects of mindfulness-based stress reduction, progressive muscle relaxation and physiotherapy. At baseline (T0; before therapy commencement) and after treatment (T1), patients were asked to complete multiple self-report questionnaires assessing socio-demographics, tinnitus-related distress, tinnitus localization, quality, frequency and loudness, as well as physical and mental health status, including levels of depression and perceived stress. All patients gave informed written consent prior to data collection. The study has been approved by the ethics committee of the Charité Universitaetsmedizin Berlin.

In total, we used data from seven questionnaires: (a) General Depression Scale-long form (Allgemeine Depressionsskala; ADSL; Radloff, 1977; Hautzinger and Bailer, 2002), (b) Perceived Stress Questionnaire (PSQ; Fliege et al., 2005), (c) Short Form-8 Health Survey (SF8; Bullinger and Morfeld, 2008), (d) a sociodemographics questionnaire (SOZK; Brueggemann et al., 2016), (e) the German version of the Tinnitus Questionnaire (TQ; Goebel and Hiller, 1998), (f) visual analogue scales measuring tinnitus loudness, frequency, and impairment (TINSKAL), and (g) the Tinnitus Localization and Quality Questionnaire (TLQ; Goebel and Hiller, 1992). These questionnaires were selected to obtain a comprehensive assessment of tinnitus, comorbid conditions (e.g., depressivity), general quality of life and sociodemographic data. Most questionnaire items comprised multiple-choice questions with answers on a Likert scale. The corresponding ordinal variables were handled as continuous variables in the analysis. Categorical variables, as reported sex, marital status, and education level were encoded as dummy variables. For data analysis, a total of 181 variables from the baseline measurements were used as predictors, including the answers to single questionnaire items, subscale scores, total scores and, for each questionnaire, the average time needed to fill in an item. A brief overview of all variables is provided in Table S1.

Tinnitus-related distress was measured by the TQ total score (TQ_distress). Depression severity was obtained using the ADSL depression score (ADSL_depression). For TQ_distress and ADSL_depression, treatment effects were calculated as differences between T0 and T1 (denoted as teffect_TQ_distress and teffect_ADSL_depression). Hence, a negative (positive) score indicates that the patient exhibited a lower (higher) score in T1 (after therapy) than in T0 (before therapy).

**Figure 1:**
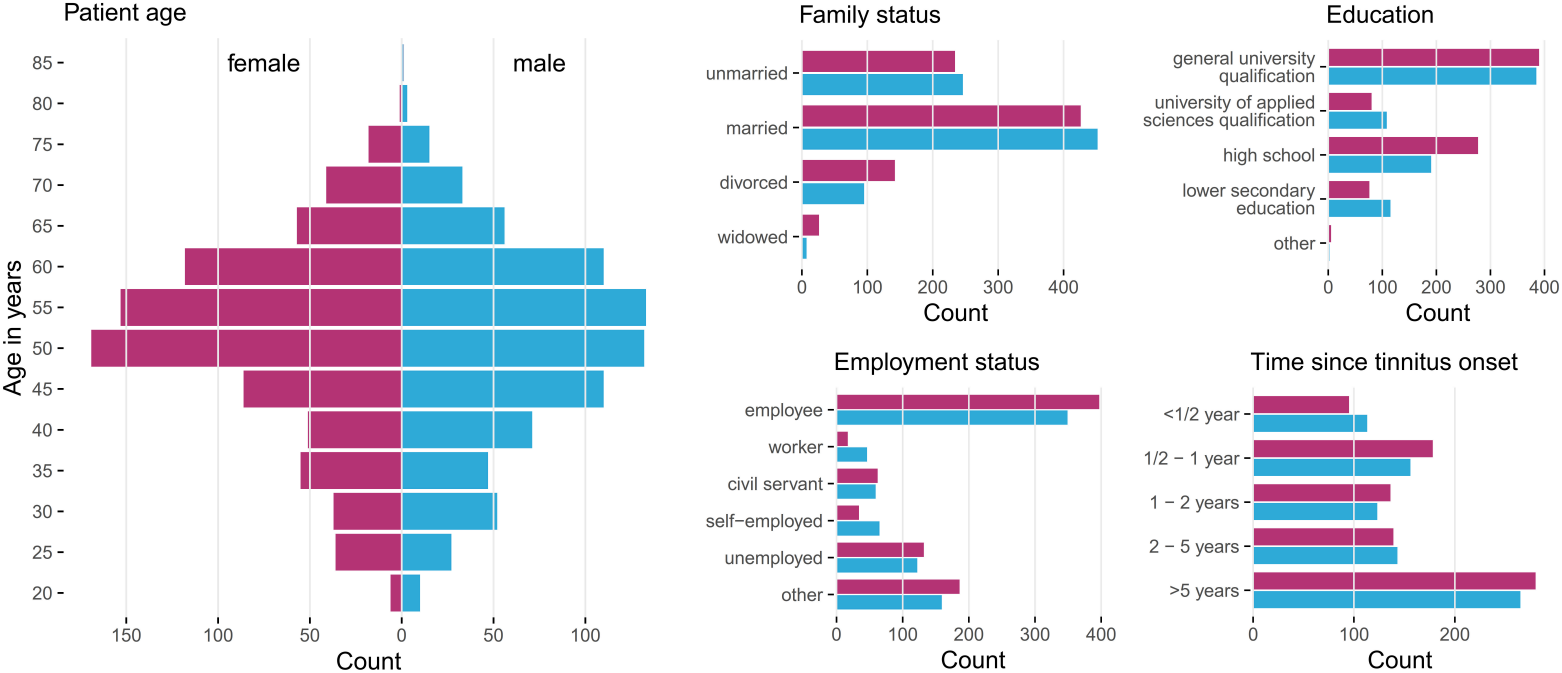
Patient characteristics by gender. Histograms summarizing basic socio-demographic properties and years since tinnitus onset of the patients stratified by gender. Data from 828 female (50.9%) and 800 male (49.1%) patients with chronic tinnitus were considered for data analysis.

Patients with incomplete data were excluded, resulting in a total number of 1,628 patients, with clinical data summarized in Figure 1. These 828 female (50.9%) and 800 male (49.1%) patients were between 18 and 83 years of age, with an average of 50.2 years (SD = 12.0). Approximately a third (33%) had been suffering from tinnitus (bilateral: 41%, unilateral left: 32%, unilateral right: 26%) for more than 5 years.

### 2.2. Data Analysis Workflow

Our data analysis workflow consisted of three components, (i) building machine learning models that are capable of separating between female and male patients and building gender-specific models with either tinnitus-related distress, depression severity, or treatment effect of each of these two as target variable, (ii) tuning the models via a hyperparameter grid in a cross-validation scheme, and (iii) identifying variables (questionnaire answers) that contributed most to the models' predictive performances, i.e., were highly associated with the target variables.

#### 2.2.1. Building Classification and Regression Models

We used machine learning classification and regression algorithms to train models on gender, tinnitus-related distress, depression, and treatment response. More specifically, we defined five *learning tasks* (LT) for the following target variables (dependent variables) and data subsets, respectively:

- LT1—target: gender; all patients
- LT2—target: TQ_distress (tinnitus distress) in T0; LT2a: female patients; LT2b: male patients
- LT3—target: ADSL_depression (depression) in T0; LT3a: female patients; LT3b: male patients
- LT4—target: teffect_TQ_distress (treatment effect); LT4a: female patients; LT4b: male patients
- LT5—target: teffect_ADSL_depression; LT5a: female patients; LT5b: male patients.

LT1 has the goal of separating the data on gender and identifying the questionnaire answers and scores that contribute to this separation, i.e., are characteristic of one of the genders. For every learning task, we used variables from baseline (T0) as predictors. Furthermore, we removed variables derived from the same questionnaire as the task's target variable. For example, for LT2, all variables from the TQ questionnaire were excluded since the target variable was calculated from them. The learning tasks LT2, LT3, LT4, and LT5 build models over female patients only and models over male patients only. We denote these models as “F_model,” resp. “M_model” hereafter; the learning task is given explicitly, if it cannot be concluded from the context.

We utilized the following five learning algorithms: least absolute shrinkage and selection operator (LASSO; Friedman et al., 2010), RIDGE (Hoerl and Kennard, 1970), support vector machine (SVM; Boser et al., 1992), random forest (RF; Breiman, 2001), and gradient boosted trees (GBT; Friedman, 2001).

#### 2.2.2. Model Evaluation and Parameter Tuning

We used 10-fold stratified cross-validation to evaluate model generalization performance. In k-fold cross-validation, the data is randomly split into k approximately equally-sized partitions. The performance of a model trained on (k-1) partitions is measured by the remaining partition which serves as test set. Finally, the k single performance results are averaged into an overall estimate. For some algorithms, the predictive performance of their models depend substantially on the selection of appropriate hyperparameter values. Therefore, we employed a grid search for hyperparameter selection (cf. listing of parameter candidates in Table S2).

For LT1, we used accuracy as primary performance measure and sensitivity as secondary performance measure. Accuracy measures the number of observations that were correctly classified by the model. Sensitivity quantifies the true *positive* rate, i.e., the number of positive observations that were correctly classified as positive. We calculated sensitivity for each gender, respectively. For the remaining learning tasks with quantitative target variables, root mean squared error (RMSE) served as primary performance measure, defined as $RMSE=\sqrt{\frac{1}{N}\overset{N}{\underset{i=1}{\sum }}{\left({ŷ}_{i}-y_{i}\right)}^{2}}$, where *N* is the total number of observations, ŷ_*i*_ is the predicted and *y*_*i*_ is the actual value of the target variable for observation *i*. The secondary performance measure was the coefficient of determination *R*^2^, defined as $R^{2}=1-\frac{\overset{N}{\underset{i=1}{\sum }}{\left({ŷ}_{i}-y_{i}\right)}^{2}}{\overset{N}{\underset{i=1}{\sum }}{\left(ȳ-y_{i}\right)}^{2}}$, where ȳ is the average predicted value of the model. Higher values are better for all measures except RMSE. Each model was compared against a *baseline*. For the classification problem of LT1, the baseline model always predicted the majority class over all training observations. Similarly, for the regression problems of LT2-5, the average value of a target variable over all training observations was used as prediction for every test instance.

#### 2.2.3. Variable Importance

For each learning task, individual variable attributions toward model prediction were computed using model reliance (MR; Fisher et al., 2018). MR is a permutation-based variable importance measure that calculates the increase in model error when the values of the variable of interest are randomly shuffled within the training set. Suppose *f* is a variable, *m* is a model, *e*_*orig*_ is the model error on the original training data and *e*_*perm*_ is the model error on the training data where the values of *f* are randomly permuted. Then, the model reliance *MR* is defined as ratio of $MR\left(f,m\right)=\frac{e_{perm}}{e_{orig}}$. If *f* is important for the prediction of *m*, *MR*(*f, m*) > 1. If random permutation of the feature values leads to a higher performance of the model, then the feature's attribute to model quality is low (whereupon *MR* < 1).

**Figure 2:**
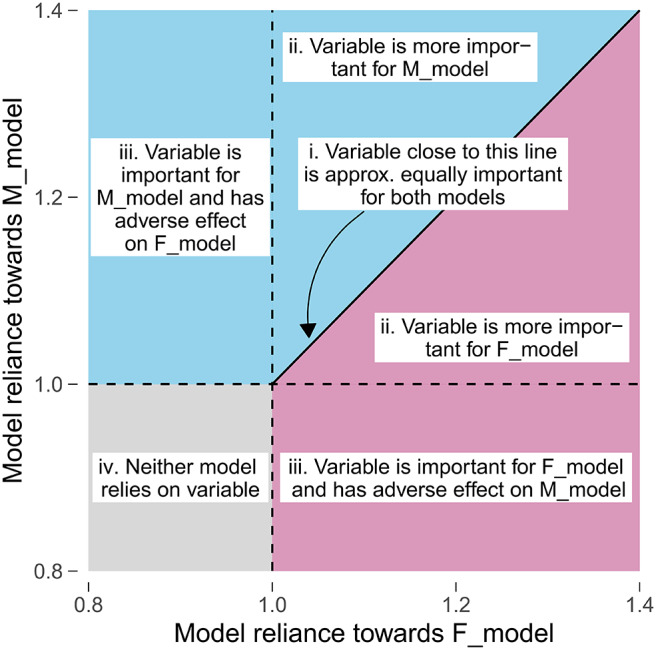
Comparison of gender-specific variable importance. The position of a point represents the model reliance score of a variable with respect to the best model trained on the female patient subset (F_model; x-axis) and the best model trained on the male patient subset (M_model; y-axis). Higher values represent a higher attribution of a variable toward the model prediction. There are four characteristic areas: (i) important variables with similar attributions toward F_model and M_model; (ii) important variables with higher attribution toward one of the gender-specific models; (iii) variables that are important for *either* F_model *or* M_model, but adversarial for the other model; (iv) variables that are adversarial for both models.

For LT1, we identified the questionnaire answers and scores that contributed to separation. For LT2-5, we focused on the variables (i.e., questionnaire items and scales) themselves. To highlight these variables, we used scatterplot diagrams (see Figure 2) that express the contribution of a feature (as *MR* value) to an F_model (x-axis) and M_model (y_axis): features that contribute to both models equally are on a diagonal line, while features with higher MR on the one model are far away from the diagonal. The variables with highest average *MR* score or highest magnitude in difference between gender-specific *MR* scores were highlighted with red color.

### 2.3. Statistical Analysis

Psychometric scores and target variable scores were compared between female and male patients using two-tailed Wilcoxon rank-sum test. Additionally, for treatment effects, it was checked whether the scores were significantly under 0 using left-tailed Wilcoxon rank-sum test. To summarize the distribution of each continuous target variable, median, median absolute deviation, and basic, non-parametric 95% confidence interval using bootstrap sampling (DiCiccio and Efron, 1996) with 2,000 samples was computed. For each model, it was checked whether cross-validation scores of the primary performance measure were significantly better than the ones of the baseline using independent one-tailed Student's *t*-test (right-tailed for accuracy; left-tailed for RMSE). A significance level of α = 0.05 was used for all statistical tests. All *p*-values were adjusted for multiple comparison using Benjamini-Hochberg correction.

## 3. Results

Female and male patients ware compared with total scores and subscales of TQ, PSQ, SF8, ADSL, and TINSKAL (cf. Table 1). Female patients reported higher tinnitus distress than the male patients, as measured by the TQ total score and its subscales “auditory perceptual difficulties,” “sleep disturbances,” and “somatic complaints.” Further, female patients had higher levels of stress, as measured by the PSQ total score and its subscales “worries,” “tension,” and “demand.” Men scored higher on the mental and physical component of SF8, respectively. Women showed higher levels of depression as measured by ADSL. Women also reported higher tinnitus frequency.

**Table 1:** Comparison of psychometric scores between female and male patients.

	**Total**	**Female**	**Male**	
**Scale**	**(*N* = 1,628)**	**(*n* = 828)**	**(*n* = 800)**	**p-value**
**TQ**				
Total score	38.53 ±17.18	39.95 ±16.34	37.06 ±17.89	<0.01^*^
Emotional distress	10.70 ± 5.68	10.88 ± 5.47	10.52 ± 5.88	0.136
Cognitive distress	6.82 ± 4.08	6.97 ± 3.99	6.67 ± 4.16	0.099
Psychological distress	17.53 ± 9.33	17.85 ± 9.01	17.19 ± 9.65	0.099
Intrusiveness	10.31 ± 3.67	10.49 ± 3.47	10.12 ± 3.87	0.130
Auditory perceptual difficulties	5.12 ± 3.70	5.41 ± 3.63	4.82 ± 3.74	<0.01^*^
Sleep disturbances	3.43 ± 2.54	3.74 ± 2.50	3.11 ± 2.54	<0.01^*^
Somatic complaints	2.15 ± 1.93	2.46 ± 1.94	1.82 ± 1.86	<0.01^*^
**PSQ**				
Total score	0.46 ± 0.18	0.48 ± 0.18	0.43 ± 0.19	<0.01^*^
Worries	0.39 ± 0.23	0.41 ± 0.22	0.37 ± 0.23	<0.01^*^
Tension	0.57 ± 0.23	0.61 ± 0.22	0.53 ± 0.24	<0.01^*^
Demand	0.49 ± 0.24	0.53 ± 0.24	0.46 ± 0.24	<0.01^*^
Joy	0.49 ± 0.23	0.48 ± 0.23	0.49 ± 0.23	0.182
**SF8**				
Mental component	41.34 ±12.10	40.16 ±11.80	42.55 ±12.29	<0.01^*^
Physical component	45.42 ± 9.71	44.44 ± 9.79	46.42 ± 9.53	<0.01^*^
**ADSL**				
Depressivity	17.93 ±11.60	19.33 ±11.44	16.48 ±11.59	<0.01^*^
**TINSKAL**				
Tinnitus impairment	4.99 ± 2.59	4.97 ± 2.66	5.02 ± 2.52	0.992
Tinnitus loudness	5.03 ± 2.62	4.95 ± 2.72	5.11 ± 2.51	0.486
Tinnitus frequency	8.09 ± 3.00	7.82 ± 3.24	8.37 ± 2.69	<0.01^*^

*Values are reported as mean ± SD. p-values were calculated using independent two-tailed Wilcoxon test adjusted for multiple comparison using Benjamini-Hochberg correction. Asterisks denote significant differences between female and male patients with a significance level of α = 0.05. ADSL: General Depression Scale-long form (Radloff, 1977; Hautzinger and Bailer, 2002); PSQ: Perceived Stress Questionnaire (Fliege et al., 2005); SF8: Short Form-8 Health Survey (Bullinger and Morfeld, 2008); TINSKAL: visual analogue scales measuring tinnitus loudness, frequency and impairment; TQ: German version of the Tinnitus Questionnaire (Goebel and Hiller, 1998)*.

Table 2 provide an overview of all learning tasks (LT), distributions of the target variables, and the models' predictive performances.

**Table 2:** Performance of classification and regression models for each learning task (LT).

**LT**	**Target**	**Target distribution**	**Method**	**Accuracy [*%*]**	**Sens. ♀[*%*]**	**Sens. ♂[*%*]**
1	Gender	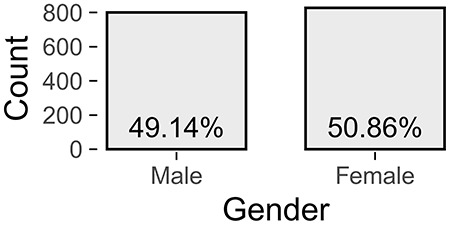	RIDGE	72.19 ± 2.94^*^	71.41 ± 5.46	73.02 ± 4.30
			SVM	71.82 ± 3.66^*^	70.93 ± 5.17	72.70 ± 6.00
			LASSO	71.33 ± 3.03^*^	69.49 ± 4.23	73.19 ± 4.88
			GBT	70.35 ± 3.36^*^	70.91 ± 5.05	69.72 ± 6.20
			RF	68.63 ± 5.24^*^	68.67 ± 5.95	68.58 ± 7.14
**LT**	**Subset**	**Target**	**Target distribution**	**Method**	**RMSE**	***R*^2^**
2a	only ♀	TQ_distress (T0)	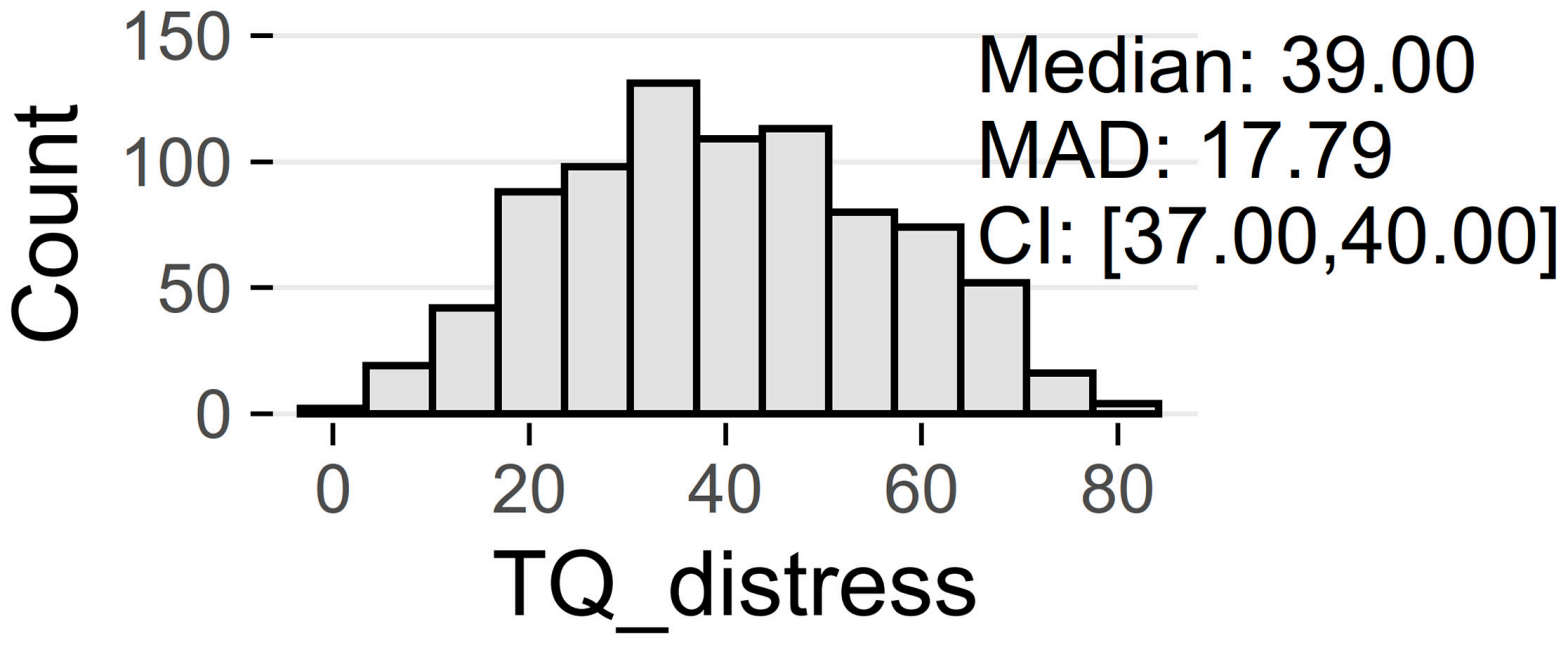	GBT	10.92 ± 0.68^*^	0.55 ± 0.04
				RF	11.38 ± 0.74^*^	0.51 ± 0.05
				LASSO	11.55 ± 0.70^*^	0.50 ± 0.04
				RIDGE	11.59 ± 0.63^*^	0.50 ± 0.04
				SVM	11.97 ± 0.51^*^	0.46 ± 0.03
2b	only ♂	TQ_distress (T0)	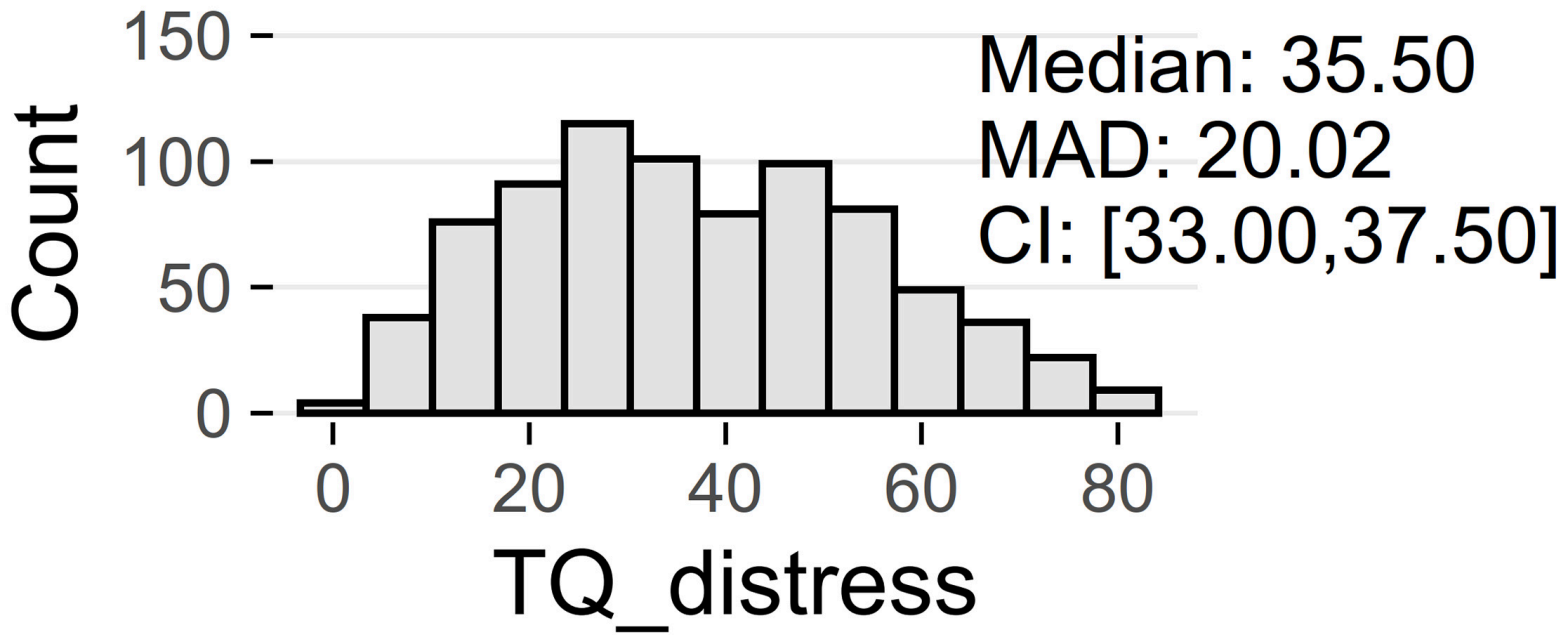	GBT	10.11 ± 1.12^*^	0.68 ± 0.06
				RF	10.58 ± 1.01^*^	0.65 ± 0.06
				LASSO	10.59 ± 0.98^*^	0.65 ± 0.05
				RIDGE	10.72 ± 1.05^*^	0.64 ± 0.06
				SVM	11.21 ± 1.02^*^	0.61 ± 0.06
3a	only ♀	ADSL_depression	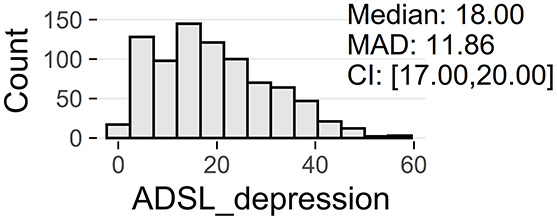	LASSO	5.80 ± 0.73^*^	0.74 ± 0.06
		(T0)		GBT	5.88 ± 0.65^*^	0.74 ± 0.06
				RF	6.02 ± 0.62^*^	0.72 ± 0.05
				GBT	6.10 ± 0.65^*^	0.72 ± 0.06
				SVM	6.12 ± 0.72^*^	0.71 ± 0.06
3b	only ♂	ADSL_depression	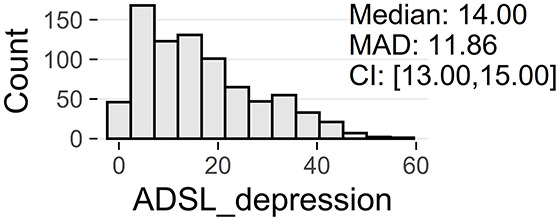	LASSO	5.10 ± 0.38^*^	0.81 ± 0.03
		(T0)		RIDGE	5.14 ± 0.38^*^	0.80 ± 0.03
				GBT	5.16 ± 0.42^*^	0.80 ± 0.04
				SVM	5.29 ± 0.38^*^	0.79 ± 0.03
				RF	5.29 ± 0.46^*^	0.79 ± 0.04
4a	only ♀	Treatment effect	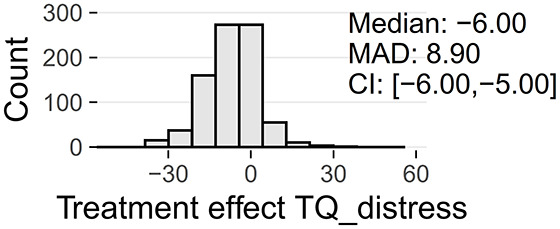	RF	9.55 ± 0.84	0.02 ± 0.09
		TQ_distress		RIDGE	9.58 ± 0.82	0.01 ± 0.09
		(T0 → T1)		GBT	9.59 ± 0.82	0.01 ± 0.08
				LASSO	9.61 ± 0.82	0.01 ± 0.08
				SVM	9.64 ± 0.83	0.00 ± 0.09
4b	only ♂	Treatment effect	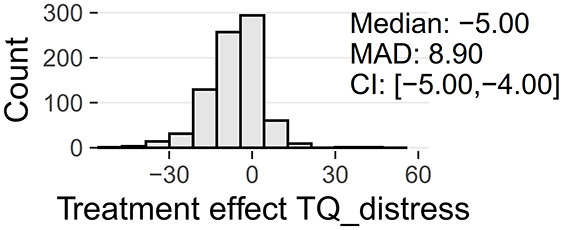	RF	9.34 ± 1.31	0.04 ± 0.14
		TQ_distress		LASSO	9.41 ± 1.34	0.03 ± 0.14
		(T0 → T1)		RIDGE	9.43 ± 1.31	0.03 ± 0.14
				GBT	9.54 ± 1.32	0.00 ± 0.14
				SVM	9.55 ± 1.36	0.00 ± 0.14
5a	only ♀	Treatment effect	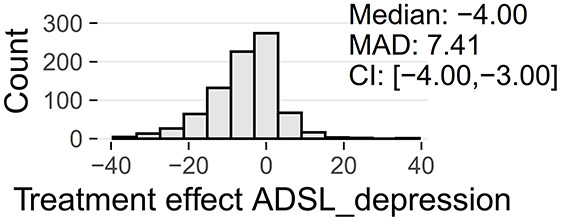	LASSO	8.21 ± 0.77	0.14 ± 0.09
		ADSL_depression		RIDGE	8.21 ± 0.80	0.14 ± 0.09
		(T0 → T1)		RF	8.23 ± 0.73	0.13 ± 0.08
				SVM	8.26 ± 0.84	0.13 ± 0.10
				GBT	8.26 ± 0.77	0.13 ± 0.09
5b	only ♂	Treatment effect	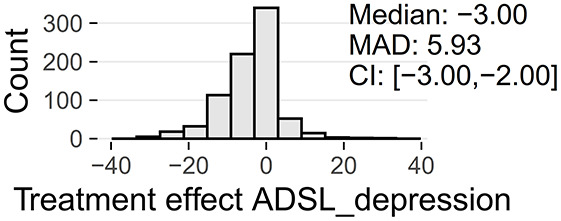	GBT	6.79 ± 0.73	0.17 ± 0.10
		ADSL_depression		RF	6.82 ± 0.68	0.16 ± 0.09
		(T0 → T1)		RIDGE	6.84 ± 0.73	0.16 ± 0.10
				LASSO	6.85 ± 0.71	0.15 ± 0.10
				SVM	7.15 ± 0.64	0.08 ± 0.09

*Performance values are cross-validation mean ± SD. Algorithms are sorted by primary performance measure, i.e., accuracy for LT1 and RMSE for LT2-5. Asterisks signify models with significantly better cross-validation performance compared to the baseline w.r.t. the primary performance measure (α = 0.05). A bar chart (LT1) and histograms (LT2-5) illustrate the distribution of the target variables. For the numerical targets, median, median absolute deviation (MAD), and 95% bootstrap confidence interval (CI) are depicted*.

### 3.1. Female Tinnitus Patients Reported Higher Levels of Tinnitus-Related Distress, Depression Severity, and Associated Treatment Effects (RQ1)

At baseline, female patients reported higher tinnitus-related distress (Median 39.00 vs. 35.50, *p* < 0.01) and depression severity (18.00 vs. 14.00, *p* < 0.01) (cf. Table 2). Using the TQ cutoff for tinnitus distress (>46; Goebel and Hiller, 1998), 34.1% of female and 30.8% of male subjects exhibited decompensated tinnitus. Using the ADSL cutoff for depression severity (>15; Hautzinger and Bailer, 2002), 57.4% of female and 45.0% of male subjects showed a clinical depression. Both patient subgroups improved from multi-modal treatment with respect to tinnitus distress: the median increase in TQ_distress score from T0 to T1 was −6.00 (*p* < 0.01) in female and −5.00 (*p* < 0.01) in male patients. For depression severity, both patient groups improved significantly (female: −4.00, *p* < 0.01; male: -3.00, *p* < 0.01). There were significant differences between female and male patients in treatment effects of tinnitus-related distress (median: −1.00, *p* = 0.04) and depression (median: −1.00, *p* < 0.01), respectively.

### 3.2. Learning Task 1: Female Patients Reported Higher Levels of Stress and Tension; Gender Differences in Tinnitus Quality (RQ2)

**Figure 3:**
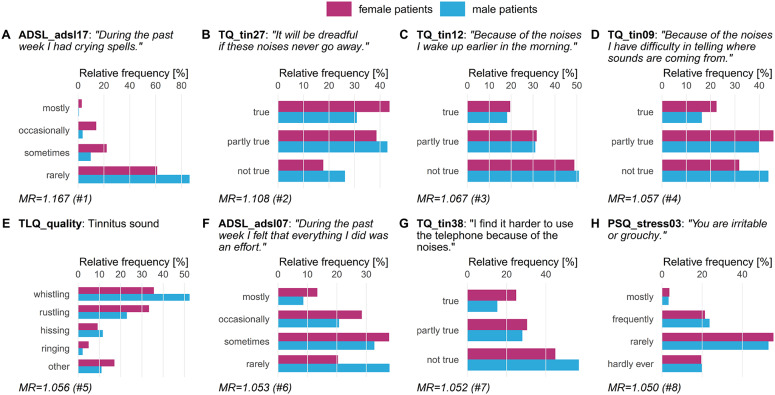
Top-8 variables on gender (LT1). Gender-stratified item answer frequencies for the top-5% variables with the highest attribution toward model prediction quality according to model reliance (MR). ADSL: General Depression Scale–long form (Radloff, 1977; Hautzinger and Bailer, 2002); PSQ: Perceived Stress Questionnaire (Fliege et al., 2005); TLQ: Tinnitus Localization and Quality Questionnaire (Goebel and Hiller, 1992); TQ: German version of the Tinnitus Questionnaire (Goebel and Hiller, 1998).

We compared the five classifiers in Table 2 (LT1) and found that RIDGE achieves best cross-validation accuracy (72.19 ± 2.94%), with a sensitivity of 71.41 ± 5.46% for female patients and 73.02 ± 4.30% for male patients. Figure 3 depicts item answer frequencies for the variables with the highest attribution toward model prediction quality: we show the variables in the top-5% of model reliance (MR). There are eight such variables, depicted in Figures 3A–E, ordered by MR value. For each variable, the horizontal legend depicts the text of the questionnaire item corresponding to this variable and the vertical axis shows the answers to this item. The horizontal axis shows the relative frequency per gender. These frequencies are shown as horizontal bars, red-violet for female patients and blue for male ones. A difference in the length of the two bars for the same answer means that the likelihood of giving this answer was different for each gender, and thus it contributes to class separation. ADSL_adsl17 (Figure 3A) was found to be the most discriminatory variable for the model (MR = 1.167): whereas 16% of female patients reported to had crying spells either “mostly” or “occasionally” during the previous week, the same number was just 4% in male patients, who predominantly gave the answer “rarely” (86.2%). Female patients tended to express higher levels of worries (cf. Figures 3B,F) and subjective stress (cf. Figure 3H). Furthermore, differences in tinnitus quality were found: more than half (52.4%) of all male patients reported the tinnitus sound (MR = 1.056) to be “whistling,” which was considerably higher than in female patients (35.6%) who described their tinnitus as a “rustling” noise more often (33.3%) than male patients (22.9%).

### 3.3. Learning Task 2: Variables Measuring Depression, Sleep Problems, Tinnitus Frequency, and Loudness Were Associated With Tinnitus-Related Distress in Both Genders (RQ3)

**Figure 4:**
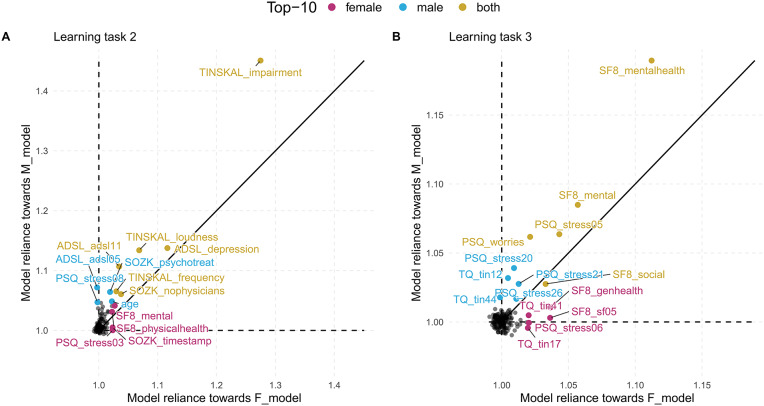
Variable importance for models on tinnitus-related distress and depression. The position of a point represents the model reliance (MR) score of a variable with respect to the best model trained on the female patient subset (F_model; x-axis) and the best model trained on the male patient subset (M_model; y-axis) (cf. Figure 2). Variables among the top-10 highest-ranked variables according to MR in the F_model, M_model, or in both models are highlighted by color. **(A)** target variable: tinnitus-related distress; **(B)** target variable: depression severity. ADSL: General Depression Scale–long form (Radloff, 1977; Hautzinger and Bailer, 2002); PSQ: Perceived Stress Questionnaire (Fliege et al., 2005); TLQ: Tinnitus Localization and Quality Questionnaire (Goebel and Hiller, 1992); TQ: German version of the Tinnitus Questionnaire (Goebel and Hiller, 1998).

We ran the five algorithms once for the female patients (block LT2a in Table 2) and once for the male patients (block LT2b in Table 2), classifying on tinnitus distress at baseline. Table 2 shows RMSE and *R*^2^ for each algorithm. For LT2a (female patients) and LT2b (male patients), GBT had best performance with respect to RMSE (LT2a: 10.92 ± 0.68, LT2b: 10.11 ± 1.12) and *R*^2^ (LT2a: 0.55 ± 0.04, LT2b: 0.68 ± 0.06). We found that GBT and all other models were slightly more accurate for male patients than for female ones. The highest variable attribution according to model reliance (MR) was achieved by TINSKAL_impairment, tinnitus impairment measured by the TINSKAL visual analogue scale, with higher attribution toward the model on male patients (MR = 1.42 vs. 1.24; cf. Figure 4A). Further, the variables ADSL_depression (depressivity), ADSL_adsl11 (sleep problems), and TINSKAL_loudness (tinnitus loudness) were found to be important for both models. Only 8 out of all 120 variables were found with a substantial attribution of *MR* > 1.05 for either of the gender-specific models.

### 3.4. Learning Task 3: Indicators of Mental Health, Stress, and Worries Associated With Depression in Both Genders (RQ3)

For depression severity at T0 (cf. Table 2 block LT3a and 3b), LASSO yielded the best model for both female patients (RMSE = 5.80 ± 0.73; *R*^2^ = 0.74 ± 0.06) and male patients (RMSE = 5.10 ± 0.38; *R*^2^ = 0.81 ± 0.03). Similarly to LT2, RMSE, and *R*^2^ estimates were better for the models on the subset of male patients. Figure 4B shows that SF8_mentalhealth (mental health score) was the most important predictor for both F_model and M_model. Further, indicators of subjective stress (PSQ_stress05: “You feel lonely or isolated.”), worries (PSQ_worries) and vitality (SF8_sf05: “During the past 4 weeks, how much energy did you have?”) contributed considerably to prediction of both genders' models.

### 3.5. Learning Tasks 4 and 5: Treatment Effect Cannot Be Reliably Predicted Using Baseline Data Only

Models trained to predict treatment effects yielded low generalization performance. Comparison of the regression algorithms in Table 2 (LT4a and LT4b) showed that the random forest model performed best predicting treatment effect on tinnitus-related distress in female patients (RMSE = 9.55 ± 0.84) as well as in male patients (RMSE = 9.34 ± 1.31). For the prediction of the treatment effect of depression LASSO (LT5a; RMSE = 8.21 ± 0.77) and GBT (LT5b; RMSE = 6.79 ± 0.73) performed best. All four models were just marginally better than the second-best models, respectively (Table 2). As for the other learning tasks, model performance on the male subset was better.

While all models of LT 1, 2a, 2b, 3a, and 3b outperformed the baselines, neither of the models predicting the treatment effect of tinnitus-related distress (LT4a and LT4b) or depressivity (LT5a and LT5b) was significantly better than the baselines. Hence, due to the poor predictive quality, we decided not to study variable attributions of these models further.

## 4. Discussion

We developed a data-driven workflow for the identification of gender-specific differences in patients with chronic tinnitus, solely based on self-report questionnaires measuring tinnitus-related distress, tinnitus frequency, loudness, localization and quality, physical and mental health status, depression severity, perceived stress, and socio-demographics that were extracted at baseline. In agreement with the findings of Seydel et al. (2013), female patients exhibited higher degrees of tinnitus distress and levels of stress than male patients. Although the trained classification models were not robust enough to separate male and female patients with very high confidence (accuracy of best model was 72%), we identified several features with a high attribution toward the model and significant differences between female and male patients, including levels of subjective stress, mood-related difficulties and tension which were higher in female patients, confirming the findings of Hébert et al. (2012), Seydel et al. (2013), and Schlee et al. (2017). Tinnitus quality was different between the genders: where the absolute majority of male patients reported their tinnitus to be a “whistling” sound more variation was observed in female patients. Notably, errors of models trained on the male subgroup were consistently lower across all learning tasks, possibly partly due to a higher variability in answers to questionnaire items in female patients. Gender-specific models exhibited similar indicators of tinnitus-related distress and depression, respectively. In particular, variables measuring similar constructs appeared to be highly relevant, e.g., the TINSKAL visual analogue scale score for tinnitus-related distress and the SF8 mental health score for depression severity. However, there were also differences in the description of tinnitus-related distress and stress experience.

In a previous study (Niemann et al., 2020), we investigated tinnitus-related distress without explicitly stratifying by gender. In agreement to the present study, TINSKAL_impairment as surrogate for TQ_distress and ADSL_depression were found to be the most important variables, gender-independent and specifically for each gender. As gender was not identified among the strongest predictors (Niemann et al., 2020), but possibly masked by other variables, we have emphasized in the present study on differences between female and male tinnitus patients on perceived tinnitus-related distress and depression. Seydel et al. (2013) provided evidence that both gender and age are the most important factors to describe tinnitus-related distress. We have confirmed differences between female and male patients with respect to tinnitus distress, tinnitus frequency, stress, mental, and physical health (cf. Table 1, Figure 3). For tinnitus-related distress as target variable, age exhibited the 9th (12th) highest model reliance score for the best male (female) model. Since our hypothesis-free approach did not pre-select any variables, we were able to identify other important variables such as TINSKAL_impairment for tinnitus-related distress and SF8_mentalhealth for depression severity.

A potential response bias [e.g., a social desirability bias (Kloostra et al., 2015; Strumila et al., 2017)], subjective judgment and differences in the experience of disorders may explain gender inconsistencies regarding self-assessment of tinnitus distress. These differences should always be considered while inspecting of results from questionnaires and may justify the development of gender-specific treatment strategies in the context of an individualized therapy. Gender differences in treatment effect with respect to tinnitus-related distress and depression severity were small but significant, despite the uniform therapy program. The treatment effect on tinnitus-related distress or depression severity could not be reliably predicted based on measurements in T0 only. Hence, future studies could investigate the potential of modular treatment procedures that combine basic, gender-specific, and individual components.

## Data Availability Statement

The datasets for this article are not publicly available because: no consent of the patients to publish their data was obtained. Requests to access the datasets should be directed to Birgit Mazurek [ birgit.mazurek@charite.de].

## Ethics Statement

The studies involving human participants were reviewed and approved by ethics committee of the Charité Universitaetsmedizin Berlin (https://ethikkommission.charite.de/en/). The patients/participants provided their written informed consent to participate in this study.

## Author Contributions

UN designed and performed the data analysis and wrote the original draft. BB, PB, and BM curated the datasets. BB, PB, BM, and MS supervised the data analysis and reviewed and edited the manuscript. BM and MS led the project.

## Conflict of Interest

The authors declare that the research was conducted in the absence of any commercial or financial relationships that could be construed as a potential conflict of interest.
