# Supplementary material for: Gender-Specific Differences in Patients With Chronic Tinnitus—Baseline Characteristics and Treatment Effects
Source: Front Neurosci. 2020 May 25;14:487. doi: 10.3389/fnins.2020.00487 (PMC7261931; doi:10.3389/fnins.2020.00487)
Supplement: Supplementary file 1 [file Data_Sheet_1.pdf]

frontiers

Supplementary Material

Table S1. Variables overview. A listing of all 181 variables that were used for modeling. These variables were extracted from item answers, subscale scores and total scores of 7 questionnaires: (a) General Depression Scale - long form (Allgemeine Depressionsskala; ADSL; Radloff (1977); Hautzinger and Bailer (2003)), (b) Perceived Stress Questionnaire (PSQ; Fliege et al. (2005)), (c) Short Form-8 Health Survey (SF8; Bullinger and Morfeld (2008)), (d) a sociodemographics questionnaire (SOZK; Brueggemann et al. (2016)), (e) the German version of the Tinnitus Questionnaire (TQ; Goebel and Hiller (1998)), (f) visual analogue scales measuring tinnitus loudness, frequency and impairment (TINSKAL) and (g) the Tinnitus Localization and Quality Questionnaire (TLQ; Goebel and Hiller (1992)).

#	Variable	Description	Response options
1	ADSL_lie	Lie detection score	-
2	ADSL_depression	Depressive disorder sum score	-
3	ADSL_adsl01	"During the past week I was bothered by things that usually don't bother me."	0=rarely; 1=sometimes; 2=occasionally; 3=mostly
4	ADSL_adsl02	"During the past week I did not feel like eating; my appetite was poor."	0=rarely; 1=sometimes; 2=occasionally; 3=mostly
5	ADSL_adsl03	"During the past week I felt that I could not shake off the blues even with help from my family or friends."	0=rarely; 1=sometimes; 2=occasionally; 3=mostly
6	ADSL_adsl04	"During the past week I felt I was just as good as other people."	0=most; 1=occasionally; 2=some; 3=rarely
7	ADSL_adsl05	"During the past week I had trouble keeping my mind on what I was doing."	0=rarely; 1=sometimes; 2=occasionally; 3=mostly
8	ADSL_adsl06	"During the past week I felt depressed."	0=rarely; 1=sometimes; 2=occasionally; 3=mostly
9	ADSL_adsl07	"During the past week I felt that everything I did was an effort."	0=rarely; 1=sometimes; 2=occasionally; 3=mostly
10	ADSL_adsl08	"During the past week I felt hopeful about the future."	0=most; 1=occasionally; 2=some; 3=rarely
11	ADSL_adsl09	"During the past week I thought my life had been a failure."	0=rarely; 1=sometimes; 2=occasionally; 3=mostly
12	ADSL_adsl10	"During the past week I felt fearful."	0=rarely; 1=sometimes; 2=occasionally; 3=mostly
13	ADSL_adsl11	"During the past week my sleep was restless."	0=rarely; 1=sometimes; 2=occasionally; 3=mostly
14	ADSL_adsl12	"During the past week I was happy."	0=most; 1=occasionally; 2=some; 3=rarely
15	ADSL_adsl13	"During the past week I talked less than usual."	0=rarely; 1=sometimes; 2=occasionally; 3=mostly
16	ADSL_adsl14	"During the past week I felt lonely."	0=rarely; 1=sometimes; 2=occasionally; 3=mostly
17	ADSL_adsl15	"During the past week people were unfriendly."	0=rarely; 1=sometimes; 2=occasionally; 3=mostly
18	ADSL_adsl16	"During the past week I enjoyed life."	0=most; 1=occasionally; 2=some; 3=rarely

Continued on next page

#	Variable	Description	Response options
19	ADSL_adsl17	"During the past week I had crying spells."	0=rarely; 1=sometimes; 2=occasionally; 3=mostly
20	ADSL_adsl18	"During the past week I felt sad."	0=rarely; 1=sometimes; 2=occasionally; 3=mostly
21	ADSL_adsl19	"During the past week I felt that people disliked me."	0=rarely; 1=sometimes; 2=occasionally; 3=mostly
22	ADSL_adsl20	"During the past week I could not get going."	0=rarely; 1=sometimes; 2=occasionally; 3=mostly
23	ADSL_timestamp	ADSL: avg. time needed to fill an item (in secs)	-
24	PSQ_demand	Demand score	-
25	PSQ_tension	Tension score	-
26	PSQ_joy	Joy score	-
27	PSQ_stress	Stress sum score	-
28	PSQ_worries	Worries score	-
29	PSQ_stress01	"You feel rested."	1=hardly ever; 2=rarely; 3=frequently; 4=mostly
30	PSQ_stress02	"You feel that too many demands are being made on you."	1=hardly ever; 2=rarely; 3=frequently; 4=mostly
31	PSQ_stress03	"You are irritable or grouchy."	1=hardly ever; 2=rarely; 3=frequently; 4=mostly
32	PSQ_stress04	"You have too many things to do."	1=hardly ever; 2=rarely; 3=frequently; 4=mostly
33	PSQ_stress05	"You feel lonely or isolated."	1=hardly ever; 2=rarely; 3=frequently; 4=mostly
34	PSQ_stress06	"You find yourself in situations of conflict."	1=hardly ever; 2=rarely; 3=frequently; 4=mostly
35	PSQ_stress07	"You feel you're doing things you really like."	1=hardly ever; 2=rarely; 3=frequently; 4=mostly
36	PSQ_stress08	"You feel tired."	1=hardly ever; 2=rarely; 3=frequently; 4=mostly
37	PSQ_stress09	"You fear you may not manage to attain your goals."	1=hardly ever; 2=rarely; 3=frequently; 4=mostly
38	PSQ_stress10	"You feel calm."	1=hardly ever; 2=rarely; 3=frequently; 4=mostly
39	PSQ_stress11	"You have too many decisions to make."	1=hardly ever; 2=rarely; 3=frequently; 4=mostly
40	PSQ_stress12	"You feel frustrated."	1=hardly ever; 2=rarely; 3=frequently; 4=mostly
41	PSQ_stress13	"You are full of energy."	1=hardly ever; 2=rarely; 3=frequently; 4=mostly
42	PSQ_stress14	"You feel tense."	1=hardly ever; 2=rarely; 3=frequently; 4=mostly

Continued on next page

#	Variable	Description	Response options
43	PSQ_stress15	"Your problems seem to be piling up."	1=hardly ever; 2=rarely; 3=frequently; 4=mostly
44	PSQ_stress16	"You feel you're in a hurry."	1=hardly ever; 2=rarely; 3=frequently; 4=mostly
45	PSQ_stress17	"You feel safe and protected."	1=hardly ever; 2=rarely; 3=frequently; 4=mostly
46	PSQ_stress18	"You have many worries."	1=hardly ever; 2=rarely; 3=frequently; 4=mostly
47	PSQ_stress19	"You are under pressure from other people."	1=hardly ever; 2=rarely; 3=frequently; 4=mostly
48	PSQ_stress20	"You feel discouraged."	1=hardly ever; 2=rarely; 3=frequently; 4=mostly
49	PSQ_stress21	"You enjoy yourself."	1=hardly ever; 2=rarely; 3=frequently; 4=mostly
50	PSQ_stress22	"You are afraid for the future."	1=hardly ever; 2=rarely; 3=frequently; 4=mostly
51	PSQ_stress23	"You feel you're doing things because you have to not because you want to."	1=hardly ever; 2=rarely; 3=frequently; 4=mostly
52	PSQ_stress24	"You feel criticised or judged."	1=hardly ever; 2=rarely; 3=frequently; 4=mostly
53	PSQ_stress25	"You are lighthearted."	1=hardly ever; 2=rarely; 3=frequently; 4=mostly
54	PSQ_stress26	"You feel mentally exhausted."	1=hardly ever; 2=rarely; 3=frequently; 4=mostly
55	PSQ_stress27	"You have trouble relaxing."	1=hardly ever; 2=rarely; 3=frequently; 4=mostly
56	PSQ_stress28	"You feel loaded down with responsibility."	1=hardly ever; 2=rarely; 3=frequently; 4=mostly
57	PSQ_stress29	"You have enough time for yourself."	1=hardly ever; 2=rarely; 3=frequently; 4=mostly
58	PSQ_stress30	"You feel under pressure from deadlines."	1=hardly ever; 2=rarely; 3=frequently; 4=mostly
59	PSQ_timestamp	PSQ: avg. time needed to fill an item (in secs)	-
60	SF8_bodily_health	Bodily pain score	-
61	SF8_genhealth	General health score	-
62	SF8_mental	Mental health summary score	-
63	SF8_mentalhealth	Mental health score	-
64	SF8_physicalhealth	Physical health summary score	-
65	SF8_physicalfunc	Physical functioning score	-
66	SF8_emotional	Role emotional score	-
67	SF8_physical	Role physical score	-
68	SF8_social	Social functioning score	-

Continued on next page

#	Variable	Description	Response options
69	SF8_sf01	"Overall, how would you rate your health during the past week?"	1=excellent; 2=very good; 3=good; 4=fair; 5=poor; 6=very poor
70	SF8_sf02	"During the past 4 weeks, how much did physical health problems limit your physical activities (such as walking or climbing stairs)?"	1=not at all; 2=very little; 3=somewhat; 4=quite a lot; 5=could not do physical activities
71	SF8_sf03	"During the past 4 weeks, how much difficulty did you have doing your daily work, both at home and away from home, because of your physical health?"	1=none at all; 2=a little bit; 3=some; 4=quite a lot; 5=could not do daily work
72	SF8_sf04	"How much bodily pain have you had during the past 4 weeks?"	1=none; 2=very mild; 3=mild; 4=moderate; 5=severe; 6=very severe
73	SF8_sf05	"During the past 4 weeks, how much energy did you have?"	1=very much; 2=quite a lot; 3=some; 4=a little; 5=none
74	SF8_sf06	"During the past 4 weeks, how much did your physical health or emotional problems limit your usual social activities with family or friends?"	1=not at all; 2=very little; 3=somewhat; 4=quite a lot; 5=could not do social activities
75	SF8_sf07	"During the past 4 weeks, how much have you been bothered by emotional problems (...) ?"	1=not at all; 2=slightly; 3=moderately ately; 4=quite a lot; 5=extremely
76	SF8_sf08	"During the past 4 weeks, how much did personal or emotional problems keep you from doing your usual work, school or other daily activities?"	1=not at all; 2=very little; 3=somewhat; 4=quite a lot; 5=could not do daily activities
77	SF8_timestamp	SF8: avg. time needed to fill an item (in secs)	-
78	SF8_vitality	Vitality score	-
79	SOZK_gender	Male gender	-
80	SOZK_nationality	German nationality	-
81	SOZK_partnership	In partnership	-
82	SOZK_divorced	Divorced	-
83	SOZK_married	Married	-
84	SOZK_unmarried	Unmarried	-
85	SOZK_abitur	Education level: "Abitur"	-
86	SOZK_fachabitur	Education level: "Fachabitur"	-
87	SOZK_lowersec	Education level: lower secondary school	-
88	SOZK_mittlreife	Education level: "mittlere Reife"	-
89	SOZK_geselle	Education level: "Geselle"	-
90	SOZK_graduate	Education level: university	-
91	SOZK_nograd	Education level: none	-

Continued on next page

#	Variable	Description	Response options
92	SOZK_meister	Education level: “Meister”	-
93	SOZK_pupil	Education level: currently pupil or “Azubi”	-
94	SOZK_student	Education level: currently student	-
95	SOZK_job	Job status: currently employed	-
96	SOZK_unemp	Duration of unemployment in the last 5 years	-
97	SOZK_employee	Occupation: employee	-
98	SOZK_worker	Occupation: worker	-
99	SOZK_civservant	Occupation: civil servant	-
100	SOZK_occupother	Occupation: other	-
101	SOZK_selfempl	Occupation: self-employed	-
102	SOZK_ill	“How long have you been ill during the last 12 months? (in months)”	-
103	SOZK_tinnitusdur	“How long have you been suffering from tinnitus (in years)?”	-
104	SOZK_psychotreat	“How long have you been in psychotherapeutic treatment? (in months)”	-
105	SOZK_nophysicians	“How many physicians have you visited because of your current complaints?”	-
106	SOZK_timestamp	SOZK: avg. time needed to fill an item (in secs)	-
107	TINSKAL_impairment	Degree of tinnitus impairment	-
108	TINSKAL_frequency	Tinnitus frequency	-
109	TINSKAL_loudness	Tinnitus loudness	-
110	TINSKAL_timestamp	TINSKAL: avg. time needed to fill an item (in secs)	-
111	TLQ_timestamp	TLQ: avg. time needed to fill an item (in secs)	-
112	TLQ_rightear	The tinnitus is located in the right ear.	-
113	TLQ_leftear	The tinnitus is located in the left ear.	-
114	TLQ_bothears	The tinnitus is located in both ears.	-
115	TLQ_entirehead	The tinnitus is located in the entire head.	-
116	TLQ_whistling	Tinnitus noise: whistling	-
117	TLQ_hissing	Tinnitus noise: hissing	-
118	TLQ_ringing	Tinnitus noise: ringing	-
119	TLQ_rustling	Tinnitus noise: rustling	-
120	TQ_audpercdiff	Auditory perceptual difficulties score	-
121	TQ_cogdistress	Cognitive distress score	-
122	TQ_emodistress	Emotional distress score	-
123	TQ_intrusivness	Intrusiveness score	-
124	TQ_psychodistress	Psychological distress score	-
125	TQ_sleepdisturb	Sleep disturbances score	-
126	TQ_somaticcompl	Somatic complaints score	-
127	TQ_distress	Total tinnitus distress score	-
128	TQ_timestamp	TQ: avg. time needed to fill an item (in secs)	-
129	TQ_tin01	“I can sometimes ignore the noises even when they are there.”	0=not true; 1=partly true; 2=true

Continued on next page

#	Variable	Description	Response options
130	TQ_tin02	"I am unable to enjoy listening to music because of the noises."	0=not true; 1=partly true; 2=true
131	TQ_tin03	"It's unfair that I have to suffer with my noises."	0=not true; 1=partly true; 2=true
132	TQ_tin04	"I wake up more in the night because of my noises."	0=not true; 1=partly true; 2=true
133	TQ_tin05	"I am aware of the noises from the moment I get up to the moment I sleep."	0=not true; 1=partly true; 2=true
134	TQ_tin06	"Your attitude to the noise makes no difference to how it affects you."	0=not true; 1=partly true; 2=true
135	TQ_tin07	"Most of the time the noises are fairly quiet."	0=not true; 1=partly true; 2=true
136	TQ_tin08	"I worry that the noises will give me a nervous breakdown."	0=not true; 1=partly true; 2=true
137	TQ_tin09	"Because of the noises I have difficulty in telling where sounds are coming from."	0=not true; 1=partly true; 2=true
138	TQ_tin10	"The way the noises sound is really unpleasant."	0=not true; 1=partly true; 2=true
139	TQ_tin11	"I feel I can never get away from the noises."	0=not true; 1=partly true; 2=true
140	TQ_tin12	"Because of the noises I wake up earlier in the morning."	0=not true; 1=partly true; 2=true
141	TQ_tin13	"I worry whether I will be able to put up with this problem for ever."	0=not true; 1=partly true; 2=true
142	TQ_tin14	"Because of the noises it is more difficult to listen to several people at once."	0=not true; 1=partly true; 2=true
143	TQ_tin15	"The noises are loud most of the time.	0=not true; 1=partly true; 2=true
144	TQ_tin16	"Because of the noises I worry that there is something seriously wrong with my body."	0=not true; 1=partly true; 2=true
145	TQ_tin17	"If the noises continue my life will not be worth living."	0=not true; 1=partly true; 2=true
146	TQ_tin18	"I have lost some of my confidence because of the noises."	0=not true; 1=partly true; 2=true
147	TQ_tin19	"I wish someone understood what this problem is like."	0=not true; 1=partly true; 2=true
148	TQ_tin20	"The noises distract me whatever I am doing."	0=not true; 1=partly true; 2=true
149	TQ_tin21	"There is very little one can do to cope with the noises."	0=not true; 1=partly true; 2=true
150	TQ_tin22	"The noises sometimes give me a pain in the ear or head."	0=not true; 1=partly true; 2=true

Continued on next page

#	Variable	Description	Response options
151	TQ_tin23	"When I feel low and pessimistic the noise seems worse."	0=not true; 1=partly true; 2=true
152	TQ_tin24	"I am more irritable with my family and friends because of the noises."	0=not true; 1=partly true; 2=true
153	TQ_tin25	"Because of the noises I have tension in the muscles of my head and neck."	0=not true; 1=partly true; 2=true
154	TQ_tin26	"Because of the noises other people's voices sound distorted to me."	0=not true; 1=partly true; 2=true
155	TQ_tin27	"It will be dreadful if these noises never go away."	0=not true; 1=partly true; 2=true
156	TQ_tin28	"I worry that the noises might damage my physical health."	0=not true; 1=partly true; 2=true
157	TQ_tin29	"The noise seems to go right through my head."	0=not true; 1=partly true; 2=true
158	TQ_tin30	"Almost all my problems are caused by these noises."	0=not true; 1=partly true; 2=true
159	TQ_tin31	"Sleep is my main problem."	0=not true; 1=partly true; 2=true
160	TQ_tin32	"It's the way you think about the noise - NOT the noise itself which makes you upset."	0=not true; 1=partly true; 2=true
161	TQ_tin33	"I have more difficulty following a conversation because of the noises."	0=not true; 1=partly true; 2=true
162	TQ_tin34	"I find it harder to relax because of the noises."	0=not true; 1=partly true; 2=true
163	TQ_tin35	"My noises are often so bad that I cannot ignore them."	0=not true; 1=partly true; 2=true
164	TQ_tin36	"It takes me longer to get to sleep because of the noises."	0=not true; 1=partly true; 2=true
165	TQ_tin37	"I sometimes get very angry when I think about having the noises."	0=not true; 1=partly true; 2=true
166	TQ_tin38	"I find it harder to use the telephone because of the noises."	0=not true; 1=partly true; 2=true
167	TQ_tin39	"I am more liable to feel low because of the noises."	0=not true; 1=partly true; 2=true
168	TQ_tin40	"I am able to forget about the noises when I am doing something interesting."	0=not true; 1=partly true; 2=true
169	TQ_tin41	"Because of the noises life seems to be getting on top of me."	0=not true; 1=partly true; 2=true
170	TQ_tin42	"I have always been sensitive about trouble with my ears."	0=not true; 1=partly true; 2=true
171	TQ_tin43	"I often think about whether the noises will ever go away."	0=not true; 1=partly true; 2=true

Continued on next page

#	Variable	Description	Response options
172	TQ_tin44	“I can imagine coping with the noises.”	0=not true; 1=partly true; 2=true
173	TQ_tin45	“The noises never ’let up’.”	0=not true; 1=partly true; 2=true
174	TQ_tin46	“A stronger person might be better at accepting this problem.”	0=not true; 1=partly true; 2=true
175	TQ_tin47	“I am a victim of my noises.”	0=not true; 1=partly true; 2=true
176	TQ_tin48	“The noises have affected my concentration.”	0=not true; 1=partly true; 2=true
177	TQ_tin49	“The noises are one of those problems in life you have to live with.”	0=not true; 1=partly true; 2=true
178	TQ_tin50	“Because of the noises I am unable to enjoy the radio or television.”	0=not true; 1=partly true; 2=true
179	TQ_tin51	“The noises sometimes produce a bad headache.”	0=not true; 1=partly true; 2=true
180	TQ_tin52	“I have always been a light sleeper.”	0=not true; 1=partly true; 2=true
181	age	patient age	-

Table S2. Hyperparameter tuning grid. The table provides an overview about each algorithm, including the associated R package, tuned hyperparameters and corresponding candidate value ranges. If necessary, any other hyperparameter were set to its default value. Hyperparameter selection was conducted for five algorithms: LASSO (Friedman et al., 2010), RIDGE (Hoerl and Kennard, 1970), support vector machine (SVM; Boser et al. (1992)), random forest (RF; Breiman (2001)) and gradient boosted trees (GBT; Friedman (2001)). All classification and regression models were built with the statistical programming language R (R Core Team, 2019) using the package `mlr` (Bischl et al., 2016), which provides a consistent interface to many machine learning algorithms from other R packages. A grid search was employed for hyperparameter tuning using accuray (RMSE) as evaluation measure for classification (regression) algorithms.

Algorithm (R package)	Hyperparameter	Min	Max	#Values
LASSO, RIDGE (both `glmnet` (Friedman et al., 2010))	`lambda`	0.01	10^{10}	100
SVM (`e1071` (Meyer et al., 2019))	`cost`	0.01	3	6
	`gamma`	0	3	4
	`kernel`	*	-	4
RF (`ranger` (Wright and Ziegler, 2017))	`mtry`	4	100	7
	`min.node.size`	1	25	6
GBT (`xgboost` (Chen et al., 2019))	`eta`	0.01	0.4	4
	`max_depth`	1	3	3
	`colsample_bytree`	0.2	1	5
	`min_child_weight`	0.5	2	3
	`subsample`	0.2	1	3
	`nrounds`	50	250	3

REFERENCES

Bischl, B., Lang, M., Kotthoff, L., Schiffner, J., Richter, J., Studerus, E., et al. (2016). mlr: Machine learning in r. *Journal of Machine Learning Research* 17, 1–5

Boser, B. E., Guyon, I. M., and Vapnik, V. N. (1992). A training algorithm for optimal margin classifiers. In *Proc. of Workshop on Computational Learning Theory* (ACM), 144–152

Breiman, L. (2001). Random forests. *Machine learning* 45, 5–32

Brueggemann, P., Szczepek, A. J., Rose, M., McKenna, L., Olze, H., and Mazurek, B. (2016). Impact of multiple factors on the degree of tinnitus distress. *Frontiers in human neuroscience* 10, 341

Bullinger, M. and Morfeld, M. (2008). Der SF-36 Health Survey. In *Gesundheitsökonomische Evaluationen* (Springer). 387–402

Chen, T., He, T., Benesty, M., Khotilovich, V., Tang, Y., Cho, H., et al. (2019). *xgboost: Extreme Gradient Boosting*. R package version 0.90.0.2

Fliege, H., Rose, M., Arck, P., Walter, O. B., Kocalevent, R.-D., Weber, C., et al. (2005). The Perceived Stress Questionnaire (PSQ) reconsidered: validation and reference values from different clinical and healthy adult samples. *Psychosomatic medicine* 67, 78–88

Friedman, J., Hastie, T., and Tibshirani, R. (2010). Regularization paths for generalized linear models via coordinate descent. *Journal of Statistical Software* 33, 1–22

Friedman, J. H. (2001). Greedy function approximation: a gradient boosting machine. *Annals of Statistics* , 1189–1232

Goebel, G. and Hiller, W. (1992). Psychische Beschwerden bei chronischem Tinnitus: Erprobung und Evaluation des Tinnitus-Fragebogens (TF). *Verhaltenstherapie* 2, 13–22

Goebel, G. and Hiller, W. (1998). *Tinnitus-Fragebogen:(TF); ein Instrument zur Erfassung von Belastung und Schweregrad bei Tinnitus; Handanweisung* (hogrefe, Verlag für Psychologie)

Hautzinger, M. and Bailer, M. (2003). ADS-Allgemeine Depressionsskala. In *Diagnostische Verfahren in der Psychotherapie* (Beltz)

Hoerl, A. E. and Kennard, R. W. (1970). Ridge regression: Biased estimation for nonorthogonal problems. *Technometrics* 12, 55–67

Meyer, D., Dimitriadou, E., Hornik, K., Weingessel, A., and Leisch, F. (2019). *e1071: Misc Functions of the Department of Statistics, Probability Theory Group (Formerly: E1071), TU Wien*. R package version 1.7-3

R Core Team (2019). *R: A Language and Environment for Statistical Computing*. R Foundation for Statistical Computing, Vienna, Austria

Radloff, L. S. (1977). The CES-D scale: a self-report depression scale for research in the general population. *Applied psychological measurement* 1, 385–401

Wright, M. N. and Ziegler, A. (2017). ranger: A fast implementation of random forests for high dimensional data in C++ and R. *Journal of Statistical Software* 77, 1–17. doi:10.18637/jss.v077.i01
